# Supplementary material for: Disruption of Mitophagy Flux through the PARL-PINK1 Pathway by CHCHD10 Mutations or CHCHD10 Depletion
Source: Cells. 2023 Dec 7;12(24):2781. doi: 10.3390/cells12242781 (PMC10741529; doi:10.3390/cells12242781)
Supplement: Supplementary file 1 [file cells-12-02781-s001.zip › cells-2708200-supplementary.pdf]

Supplemental Figure Legends

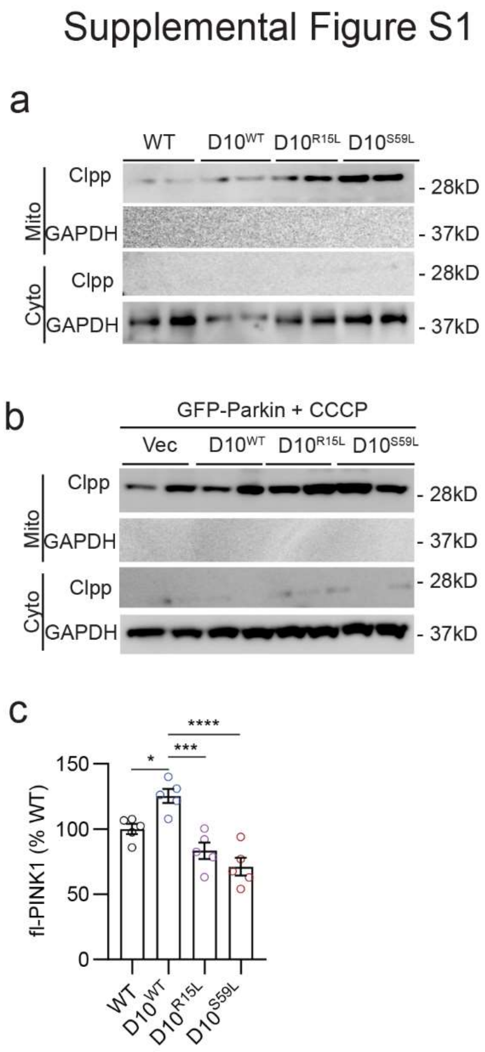

Figure S1. CHCHD10^{R15L} and CHCHD10^{S59L} mutations inhibit Parkin recruitment and reduce fl-PINK1 levels. (a) Mitochondrial and cytosolic fractions isolated from 10-month-old WT and CHCHD10 Tg mice (WT, R15L, & S59L) and immunoblotted for Clpp and GAPDH. (b) HEK293T cells co-transfected with GFP-Parkin and vector control or Flag-CHCHD10 variants (WT, R15L, & S59L), treated with CCCP (10μM, 4h), subjected to isolation of mitochondrial and cytosolic fractions, and immunoblotted for Clpp and GAPDH. (c) Total full length PINK1 (fl-PINK1) quantified from the cortex of 10-month-old WT and CHCHD10 Tg mice (WT, R15L, & S59L) (1-

way ANOVA, $F(3, 16)=16.76$, $P<0.0001$; posthoc Dunnett, *$P<0.05$, ***$P<0.001$, ***$P<0.0001$, n=5 mice/genotype). Representative blot in Figure 2a.

Supplemental Figure S2

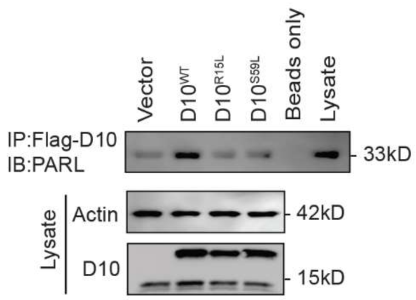

Figure S2. CHCHD10 interactions with PARL is suppressed by CHCHD10^{R15L} and CHCHD10^{S59L} mutations. Equal amount of protein from HEK293T cells transfected with vector control or Flag-CHCHD10 variants (WT, R15L, or S59L) subjected to immunoprecipitation (IP) for Flag (M2) and immunoblotted for PARL (upper panel). Lysates directly immunoblotted for Actin and CHCHD10 (lower panels).

Supplemental Figure S3

Case Number	Primary Neuropathologic Diagnosis	Braak Stage	ABC Score	CERAD Score	Thal Score	PMI (hr)	Age at Onset	Age at Death	Duration	ApoE	Race/Sex
OS99-08	Control	I	None	None	0	3		74		E3/3	wf
OS00-23	Control	0	Low	None	1	11		68		E3/3	bf
OS02-35	Control	I	None	None	0	6		75		E3/3	wf
OS03-390	Control	I	None	None	0	7		74		E3/3	wf
E04-34	Control	I	Low	None	2	17		57		E3/3	bf
E05-74	Control	I	None	None	0	6		59		E2/3	bm
E06-41	Control	II	None	None	0	10		57		E3/3	wm
E08-101	Control	II	None	None	0	11.5		78		E3/3	wf
E08-137	Control	III	None	None	0	15.5		92		E3/3	wf
E08-145	Control	0	None	None	0	28		45		E3/3	wf
E10-142	Control	II	None	None	0	5.5		94		E3/3	wm
OS01-33	FTLD-TDP	I	Low	Sparse	2	16.5	53-58	63	5 to 10	E3/3	wm
OS01-134	FTLD-TDP (C9 expansion)	II	Low	None	2	na	68	69	<1	E3/3	wf
OS03-95	FTLD-TDP/p62 (C9 negative)	0	None	None	0	21	52	59	7	E3/3	wm
E04-125	FTLD-TDP	I	None	None	0	17.5	56	61	5	E3/3	wm
E04-155	FTLD-TDP	I	Low	None	1	6	56	64	8	E3/4	wf
E05-200	FTLD-TDP	II	None	None	0	18	62	71	9	E3/3	wf
E06-160	FTLD-TDP (C9 expansion)	III	Low	None	2	6	57	66	9	E3/3	wm
E09-91	FTLD-TDP	II	Low	None	1	11.5	66	67	~1	E2/3	wm
E14-111	FTLD-TDP (C9 expansion)	IV	Low	None	1	17	60	70	10	E2/3	wf
E16-131	FTLD-TDP (C9 expansion)	III	Low	None	1	3.5	56	70	14		wf

Figure S3. Postmortem human brain case information. Case information of FTLD-TDP and nondementia control brains. Frozen frontal gyrus brain tissues were obtained from the Alzheimer's Disease Research Center (ADRD) at Emory University. Available information on APOE genotypes, ethnicity (w=White, b=Black, h=Hispanic), sex (m=male, f=female), postmortem interval (PMI), age at onset of disease, age at death, Braak stage, ABC Score, CERAD score, and Thal score are indicated.

Supplemental Figure S4

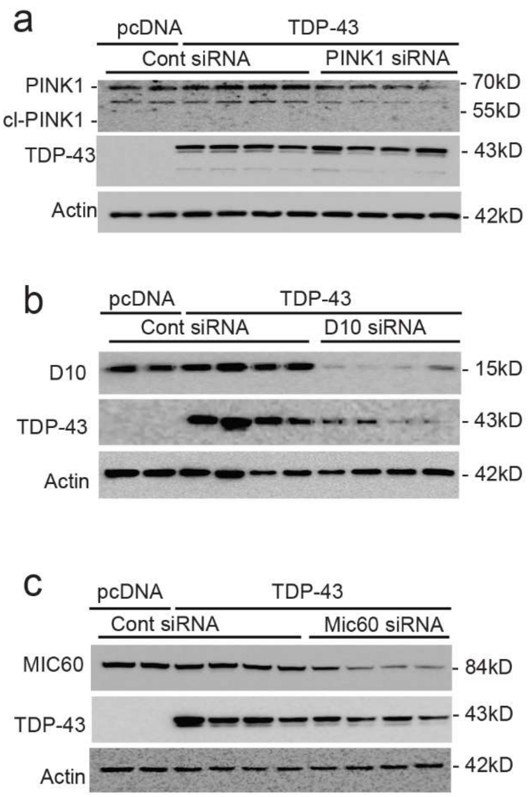

Figure S4. TDP-43, PINK1, CHCHD10, and MIC60 expression (a-c) Immunoblots for TDP-43, PINK1, CHCHD10, MIC60, and Actin from RIPA-soluble fractions used in Figure 7a with and without (a) PINK1 siRNA, (b) CHCHD10 siRNA, or (c) MIC60 siRNA.
